# Practice patterns of naturopathic physicians: results from a random survey of licensed practitioners in two US States

**DOI:** 10.1186/1472-6882-4-14

**Published:** 2004-10-20

**Authors:** Heather S Boon, Daniel C Cherkin, Janet Erro, Karen J Sherman, Bruce Milliman, Jennifer Booker, Elaine H Cramer, Michael J Smith, Richard A Deyo, David M Eisenberg

**Affiliations:** 1 Leslie Dan Faculty of Pharmacy, University of Toronto, Toronto, Ontario, Canada; 2 Center for Health Studies, Group Health Cooperative, Seattle, Washington, USA; 3 Bastyr University, Naturopathic Medicine Program, Kenmore, Washington, USA; 4 Private Practice, Olympia, Washington, USA; 5 Centers for Disease Control, Vessel Sanitation Program, Atlanta, Georgia, USA; 6 Natural Health Products Directorate, Health Canada, Ottawa, Ontario, Canada; 7 University of Washington Departments of Medicine and Health Services, Seattle, Washington, USA; 8 Beth Israel-Deaconess Center for Alternative Medicine Research and Education, Department of Medicine, Harvard Medical School, Boston, Massachusetts, USA

## Abstract

**Background:**

Despite the growing use of complementary and alternative medicine (CAM) by consumers in the U.S., little is known about the practice of CAM providers. The objective of this study was to describe and compare the practice patterns of naturopathic physicians in Washington State and Connecticut.

**Methods:**

Telephone interviews were conducted with state-wide random samples of licensed naturopathic physicians and data were collected on consecutive patient visits in 1998 and 1999. The main outcome measures were: Sociodemographic, training and practice characteristics of naturopathic physicians; and demographics, reasons for visit, types of treatments, payment source and visit duration for patients.

**Result:**

One hundred and seventy practitioners were interviewed and 99 recorded data on a total of 1817 patient visits. Naturopathic physicians in Washington and Connecticut had similar demographic and practice characteristics. Both the practitioners and their patients were primarily White and female. Almost 75% of all naturopathic visits were for chronic complaints, most frequently fatigue, headache, and back symptoms. Complete blood counts, serum chemistries, lipids panels and stool analyses were ordered for 4% to 10% of visits. All other diagnostic tests were ordered less frequently. The most commonly prescribed naturopathic therapeutics were: botanical medicines (51% of visits in Connecticut, 43% in Washington), vitamins (41% and 43%), minerals (35% and 39%), homeopathy (29% and 19%) and allergy treatments (11% and 13%). The mean visit length was about 40 minutes. Approximately half the visits were paid directly by the patient.

**Conclusion:**

This study provides information that will help other health care providers, patients and policy makers better understand the nature of naturopathic care.

## Background

The number of Americans using complementary and alternative medicine (CAM) rose from 34% to 42% from 1990 to 1997, with annual spending for CAM therapies in excess of $21 billion [1]. The majority of the research on the use of CAM services has focused on patients or consumers of CAM. Several patient characteristics have been found to be associated with increased CAM use including female gender, child-bearing age, above average income, above average education and diagnosis with a chronic or life-threatening condition [1-4]. Reasons why consumers seek care from CAM practitioners have also been identified. These include: the failure of conventional treatment to alleviate symptoms; psychological congruence between the individual's belief system and the CAM therapy; individuals' expectations that use of CAM will empower them by offering a greater sense of control over personal health care decisions; adverse effects of conventional therapies; and dissatisfaction with the care of conventional practitioners [1-3,5-8].

Although we now have some understanding of who seeks CAM care and why, relatively little is known about the actual practices of CAM practitioners including the type of patients they treat, the kinds of therapies they use, and the numbers of patients they see each day. In addition, it is unclear if the practice patterns of CAM practitioners vary by state. The objective of this paper is to describe and compare the practice of naturopathic physicians in two US states: Washington and Connecticut.

### Naturopathic medicine

Naturopathic medicine is a system of health care, based on the teachings of Benedict Lust [9], with a primary goal of enhancing the individual's innate self-healing ability by employing a variety of natural and largely non-invasive healing modalities. In some states, naturopathic physicians are authorized to employ more invasive interventions (i.e., prescription drugs, and minor surgery). Naturopathic medicine is defined by the American Association of Naturopathic Physicians (AANP) as:

... a distinct system of primary health care – an art, science, philosophy and practice of diagnosis, treatment and prevention of illness. Naturopathic medicine is distinguished by the principles upon which its practice is based. These principles are continually reexamined in the light of scientific advances. The techniques of naturopathic medicine include modern and traditional, scientific and empirical methods [10].

Naturopathic physicians are trained as generalists with expertise in a variety of core treatment methods including nutrition, hydrotherapy, colonic irrigation, physiotherapy, naturopathic manipulation, botanical medicine, homeopathy, pharmacology and minor office surgical procedures. Some licensed naturopathic physicians are also trained in traditional Chinese medicine, acupuncture and Ayurvedic medicine as well as clinical specialties such as natural childbirth [9,11-14]. There are currently six North American schools of naturopathic medicine whose graduates are eligible to sit state licensing examinations: Bastyr University (Seattle, WA), the Canadian College of Naturopathic Medicine (Toronto, ON, Canada), National College of Naturopathic Medicine (Portland, OR), Southwest College of Naturopathic Medicine (Tempe, AZ), the University of Bridgeport College of Naturopathic Medicine (Bridgeport, CT) (has applied for accreditation candidacy with the Council on Naturopathic Medical Education (CNME)), and the West Coast Naturopathic Medical College (Vancouver, BC) (not accredited by the CNME) [9].

Accurate estimates of the number of naturopathic physicians practicing in North America are difficult to obtain. A recent estimate (based on data from licensing bodies) indicated that in 2000 approximately 1300 licensed naturopathic physicians were practicing in the United States and another 500 were practicing in Canada. Naturopathic medicine is a licensed health care profession in twelve US states (Alaska, Arizona, Connecticut, Hawaii, Maine, Montana, New Hampshire, Oregon, Utah, Vermont, Washington, California), Puerto Rico and four Canadian provinces (British Columbia, Manitoba, Ontario and Saskatchewan) [9,15]. In most states and provinces where naturopathic medicine is not regulated, individuals may practice similar therapeutic approaches and/or call themselves naturopaths (whether or not they have been trained at a school for naturopathic medicine) because the term naturopathic medicine is not a restricted term. The number of individuals practicing in unregulated jurisdictions is unknown. In South Carolina and Tennessee, it is illegal to practice naturopathy [9].

Legal regulation of naturopathic medicine varies by state and province. Connecticut and Washington have relatively similar (and representative of the other ten states) requirements for licensure including: Graduation from an accredited four-year naturopathic medical school, successful completion of licensing examination (normally NPLEX), the submission of completed application forms and paying the required fee. In addition, Washington State requires that individuals be of good moral character with no history of unprofessional conduct [9].

The legal scopes of naturopathic practice in Connecticut and Washington are similar and relatively broad compared to the other states that license naturopathic physicians. Naturopathic physicians in these two states can diagnose and treat disease, utilizing a wide range of modalities and specialties including: minor surgery such as suturing (limited in Washington); physical therapies including hydrotherapy, naturopathic manipulation, and physiotherapy; colonic irrigation; electrotherapy (for example TENs); diagnostic x-ray; venipuncture; obstetrics (in Washington a midwifery license is required); gynecology; botanical medicine; nutrition; and homeopathy. Naturopathic physicians in Washington may prescribe a limited range of drugs; however, this is not part of the scope of practice in Connecticut. The practice of acupuncture requires separate licensure in both states [9].

## Methods

### Study design

The data derive from a parent study of licensed acupuncturists, chiropractors, massage therapists and naturopathic physicians [16]. The study was conducted in two phases: 1) random samples of licensed naturopathic physicians were interviewed by telephone; 2) a sub-set of those interviewed were recruited to record detailed information on 20 consecutive patient visits. Data were collected for each practitioner group in two states (one in the West and one in the Northeast). The West and Northeast were selected because these are the regions where CAM practitioners are concentrated in the United States [14,17]. The data for naturopathic physicians were collected in Washington and Connecticut.

### Sample

Licensing Boards provided contact information for all licensed naturopathic physicians in Washington (1998) and in Connecticut (1999) with in-state addresses. In total, 142 licensed practitioners were identified in Washington and 63 licensed practitioners were identified in Connecticut. Providers without identifiable telephone numbers, and those not currently practicing were excluded. The proportion of excluded providers was 11% in Connecticut and 29% in Washington. See Table 1 for additional sampling details.

**Table 1:** Selection and participation of naturopathic physicians (NDs) in Washington (1999) and Connecticut (1998)

	**Connecticut**	**Washington**
NDs licensed in state	71	286
NDs randomly selected for study	71	200
NDs found eligible for study*	63	142
Eligible NDs interviewed	59	111
Interviewed NDs eligible to collect visit data**	55	93
Eligible NDs providing visit data	34	65
Total patient visits reported	631	1186

* NDs confirmed to be practicing in state and to have verifiable and functioning phone number

** NDs who reported seeing 10 or more patients in a typical week

To maximize the accuracy of state-wide estimates, data collection efforts were concentrated on high-volume practitioners: those with at least 20 patient visits per week. About 60% to 70% of practitioners had a high-volume practice and accounted for roughly 85% to 90% of all visits to the profession. The remaining practitioners were categorized as low-volume providers. While all high-volume practitioners were asked to collect data on 20 consecutive patient visits, only the first 10 low-volume practitioners were asked to collect data. The rationale was to collect only enough data from low-volume practitioners to ascertain whether their practices differed markedly from those of high-volume practitioners. It was ultimately decided, however, to weight data for high- and low-volume practitioners in a manner that produced annual estimates of visits in each state.

Naturopathic physicians who agreed to participate were asked to complete one-page encounter forms for 20 consecutive patient visits. No financial incentives were provided for participation in the study and the protocol was approved by the Group Health Human Subjects Review Committee and the Harvard Pilgrim Health Care "Committee on Clinical Investigations, New Procedures and New Forms of Therapy" prior to the commencement of data collection.

### Data collection

Research assistants conducted telephone interviews with the randomly selected providers in Washington State in 1998 and in all eligible practitioners in Connecticut in 1999. (Given the small number of eligible practitioners in Connecticut, all were contacted). Information about individual demographic characteristics (e.g., age, gender, race/ethnicity); training characteristics (e.g., duration, location); practice characteristics (e.g., length of time in practice, licensure in other health care professions, practice arrangement); and workload characteristics (e.g., number of weeks in practice per year; hours per week of direct patient care) was obtained for each provider who agreed to be interviewed. Among those eligible to be interviewed, the participation rate was 94% in Connecticut and 78% in Washington.

Naturopathic physicians who were eligible to collect visit data (i.e., those who saw at least 20 visits per week plus a sample of those who saw at least 10 – 19 visits per week) were asked to complete encounter forms for 20 consecutive patient visits. (Only 2% of all visits were provided by naturopathic doctors with less than 10 visits per week.) Those who agreed were mailed encounter forms that were modeled after those used by the National Ambulatory Medical Care Survey (NAMCS) [18]. Each naturopathic physician was instructed to return the encounter forms by mail to the study center after completing them. Those who failed to promptly return their forms received a reminder telephone call. Among those eligible to collect visit data, 62% of naturopathic doctors in Connecticut and 70% of naturopathic doctors in Washington provided visit data.

## Analysis

Descriptive statistics (means, standard deviations, frequencies) were used to present the practitioner characteristics in Table 2. In the analyses of visit characteristics (Tables 4 and 5), each visit in the sample was weighted by the inverse of the sampling probability, reflecting both the chance that the particular provider participated and the estimated proportion of that provider's annual visits included in the study. Consequently, our results represent estimates of all visits made to naturopathic physicians in each state except for the roughly 2% of visits made to practitioners with the lowest volumes [16].

**Table 2:** Characteristics of naturopathic physicians licensed in Connecticut (1999) and Washington (1998)

	**Connecticut (n = 59) %**	**Washington (n = 111) %**
**Demographics**		
Female	58	57
White	95	94
Mean Age (SD)	43.6 (8.7) years	44.1 (9.2) years
**Education**		
Institution*:		
Bastyr University	37	80
National College	59	20
Other	3	1
Specialty training^#^	69	42
Licensed in other Health Profession*	17 (acupuncture 10%; all others less than 2%)	33 (chiropractic 8%; nursing 7%; acupuncture 6%; midwifery 5%; all others less than 2%)
**Practice solo**	51	54

*significant differences between the two states, chi square, p < 0.05)

^# ^significant differences between the two states, t-test, p < 0.05)

**Table 4:** Characteristics of visits to naturopathic physicians in Connecticut (1999) and Washington (1998)

	**Connecticut (n = 631 visits) %**	**Washington (n = 1186 visits) %**
Gender		
female	76	74
Age		
Median	43 years	42 years
0–15 years	12.8	10.9
16–34 years	16.4	20.9
35–64 years	63.0	58.5
65 years+	7.8	9.7
Race		
White	97	96
Smoking status:		
non-smokers	95	94
Type of major problem^a^		
Acute problem	22	23
Chronic problem, routine	53	56
Chronic problem, flare up	20	18
Pre/post-surgery/injury	1	1
Non-illness care	5	6
Primary reason for visit^b^		
Fatigue	6.1	6.0
Headache	4.4	3.8
Back symptoms	4.4	6.5
Skin rashes	4.2	2.5
Menopausal symptoms	3.7	2.0
Bowel function changes	3.5	2.0
Anxiety or Depression	3.2	5.1
Allergies to food, milk	3.0	3.6
Sinus symptoms	2.8	<2
Upper respiratory symptoms	2.6	<2
Cough	2.4	<2
Neck symptoms	2.3	3.3
Infectious disease	2.2	<2
Ear infection symptoms	2.2	<2
Abdominal pain/cramps	2.2	2.7
Menstrual problems	<2	2.1
Diagnosis by naturopathic physician^c^		
Menopausal disorders	4.9	3.1
Allergies	4.6	6.3
Back conditions	3.5	5.8
Fibromyalgia	3.0	2.0
Sinusitis	2.9	<2
Fatigue	2.4	2.7
Headache	2.2	3.1
Upper respiratory infections	2.0	<2
Neck conditions	<2	3.3
Depression	<2	2.6
Asthma	<2	2.2
Anxiety	<2	2.1
Dermatitis	<2	2.0
New patients	22	22
Payment		
Private insurance	60	43
Worker's compensation	0	3
Personal injury	0	3
Self-pay	37	48
Other	3	3

^a ^Because 3% to 4% indicated multiple reasons, total percentages exceed 100%

^b ^Patient report; only reporting reasons totaling 2% or more of visits

^c ^Based on ICD 9 criteria; only reporting diagnoses totaling 2% or more of visits

**Table 5:** Characteristics of visits to naturopathic physicians in Connecticut (1998) and Washington (1999)

	**Connecticut (n = 631 visits) %**	**Washington (n = 1186 visits) %**
**Diagnostics/Screen Services**:		
Examinations		
Vitals (BP, pulse, temp)	28	39
HEENT	18	15
Complete physical	13	9
Mental Status	<5	6
Imaging		
x-ray	1	2
ultrasound	1	1
Blood tests		
Complete blood count	7	10
Serum chemistry	7	9
Thyroid	3	7
Lipids panel	4	5
Allergy	4	2
Additional Tests		
Stool analysis	5	4
Urine analysis	4	2
Vitamin/Mineral	3	0
Endocrine	2	3
Allergy Skin test	1	1
TB skin test	1	0
**Therapeutics and Preventive Services:**		
Naturopathic Therapeutics		
Botanical medicine	51	43
Vitamins	41	43
Minerals	35	39
Homeopathy	29	19
Acupuncture	14	4
Allergy treatment	11	13
Glandular therapies	4	13
Physical Therapies		
Naturopathic manipulation	8	15
Physiotherapy	1	13
Hydrotherapy	4	10
Ultrasound	2	9
Mechanotherapy	2	7
Counseling/Education		
Therapeutic diet	26	36
Self-care education	17	23
Exercise therapy	9	12
Mental Health	4	6
Visit disposition *		
No follow-up planned	5	4
Return if needed	18	21
Return at specified time	77	74
Referred (% to an MD)	5 (4)	6 (4)
**Visit Duration**		
Mean, minutes	40	44
<15 minutes	1	3
15–29 minutes	14	20
30 to 44 minutes	49	31
45–59 minutes	15	18
>/=60 minutes	20	28

* Totals add to more than 100% because multiple responses were allowed

Given the large sample sizes (631 and 1186), the weighted percentages presented in the tables have small standard errors, generally between 0.5 and 2.5 percentage points and rarely exceeding 3 percentage points. As a result, moderate to large differences between the states are also statistically significant and we do not include standard errors in the tables.

## Results

### Naturopathic physician participants

The demographic characteristics of the naturopathic physicians in Washington State and Connecticut were remarkably similar (see Table 2). Just over half were female, with mean ages of about 44 years. Over 90% of naturopathic physicians identified themselves as White.

The majority of practitioners (80%) in Washington State trained at Bastyr University in Seattle, while most practitioners in Connecticut trained at National College of Naturopathic Medicine in Portland, Oregon. Although naturopathic doctors in Connecticut were more likely to report advanced specialty training (such as homeopathy), those in Washington were more likely to be licensed in another health profession. Washington naturopathic physicians were most often also licensed in chiropractic (8%), nursing (7%), acupuncture (6%) and midwifery (5%), while Connecticut naturopathic physicians were most often also licensed in acupuncture (10%). Almost one-quarter (22.5%) of naturopathic doctors licensed in Washington had completed at least one year of post-graduate residency training compared with only 8.5% of those in Connecticut. (Residency training is not required for licensure in either state.)

Both groups graduated from 4–5 year accredited full time programs of naturopathic medical education and the resulting naturopathic practice characteristics were reported to be very similar across the two states (Tables 2 and 3). Just over half of the practitioners in both states practiced solo, averaged approximately 25 hours per week providing direct patient care and saw an average of just over 30 patient visits per week. Practitioners who reported seeing less than 10 patients per week were excluded from this study.

**Table 3:** Practices of naturopathic physicians licensed in Connecticut (1999) and Washington (1998)

	**Connecticut (n = 59)**	**Washington (n = 110)**
**Median number of years in practice**	9	7
**Patient care hours in a typical week**		
Mean (S.D.)	25.8 (10.6)	24.5 (12.2)
Percentage reporting: 1–14 hours	10.2	20.0
15–24 hours	32.2	30.0
25–34 hours	39.0	26.4
35–44 hours	15.2	17.2
45+ hours	3.4	6.4
**Patient visits in a typical week**		
Mean (S.D.)	32.7 (18.9)	30.5 (24.7)
Percentage reporting: 1–19 visits	20.7	34.6
20–29 visits	31.0	21.8
30–39 visits	13.8	21.8
40–49 visits	13.8	9.1
50+ visits	20.7	12.7

### Characteristics of patients who see naturopathic physicians

As was the case for the practitioners, patients of naturopathic physicians in the two states were remarkably similar (Table 4). About 75% of patients were female, about 95% were White and non-smokers, and the mean age of the patients was 41 years. Just over 50% of visits were for chronic conditions, followed in frequency by acute problems and flare-ups of chronic problems. There was extensive overlap in the most common presenting complaints (based on ICD 9 classification) in Connecticut and Washington with fatigue, headache, back symptoms among the top four in both states. However, there was a wide variety in the presenting complaints recorded; the most common presenting complaint was identified by less that 7% of patients in both states. More than one of every five visits was made by a new patient. Overall, approximately half the visits were paid for by private insurers and a similar percentage were paid directly by the patient. Visits in Connecticut were slightly more likely to be covered by insurance (60% vs 49% of visits).

### Characteristics of naturopathic medical visits

More than 70% of visits included examinations or the ordering of diagnostic or screening tests (Table 5), most often: assessment of vital signs (including blood pressure, pulse and temperature), and Head, Eyes, Ears, Nose, Throat (HEENT) assessment (typically done for upper respiratory symptoms). The most common blood tests ordered in both states were: complete blood counts and serum chemistry. Imaging and other types of diagnostic tests were used for no more than 5% of visits in both states.

Therapeutic and preventive modalities were used in most visits (96%) in both states. The most common naturopathic therapeutics used in both states were: botanical medicines, vitamins, minerals, homeopathy and allergy treatments (Table 5). In addition, glandular therapies were prevalent in Washington (13% of visits) and acupuncture was prevalent in Connecticut (14% of visits). The only physical therapies that are part of the naturopathic scope of practice used in more than 5% of the visits were naturopathic manipulation, physiotherapy (Washington only) and hydrotherapy (Washington only). Twenty-six per cent of visits in Connecticut included counseling or education compared with 36% of visits in Washington. Most often this included discussion of a therapeutic diet.

Three-quarters of all visits in both states resulted in a recommendation that the patient return at a specified time (Table 5). The mean length of visit in the two states was similar (40–44 minutes), but there was greater variability in the length in Connecticut.

A wide range of generally similar commercially packaged products (including herbs, vitamins and minerals) were used by naturopathic physicians in the two states (Table 6). Connecticut providers recommended about 50% more than Washington providers. Almost all of the top 15 commercially packaged products used in both states were vitamins and minerals as single agents or as combinations. The most common commercially packaged products were about 1.5 to 2.0 times more likely to be used in Connecticut compared to Washington. In addition, zinc and vitamin B6 were more likely to be used by NDs in Connecticut, while protomorphogens and vitamin B12 were more commonly used in Washington. (A protomorphogen is thought to be that component of the cell chromosome that is responsible for morphogenic (forming of body and organs) determination of cell characteristics.  Theories about what protomorphogens are, as well as their role in health and disease continue to be debated.)

**Table 6:** Most common commercially packaged products used by naturopathic practitionersin Connecticut (1998) and Washington (1999)

	**Connecticut (n = 631 visits)**	**Washington (n = 1186 visits)**
Number of Products Used per visit		
Range	0 to 28	0 to 26
Median	3.0	2.0
Mean	4.1	2.7
Most Common Commercially Packaged Products^a ^(weighted percent of visits)		
Multivitamins	26.7	9.1
Digestive treatments	22.3	13.2
Magnesium	16.3	9.1
Combination vitamin and mineral	16.2	10.0
Calcium	15.8	9.3
Vitamin C	15.1	10.2
Minerals, NOS^b^	9.4	7.7
Zinc	9.0	<5
Multiminerals	8.4	7.8
Vitamins, NOS^b^	8.4	10.5
Vitamin E	7.9	5.2
Bioflavonoids	7.1	5.9
Vitamin B6	5.9	<5
Coenzyme Q10	5.8	<5
Selenium	5.2	<5
Protomorphogen	<5	9.0
Vitamin B12	<5	7.4

^a ^Excluding diets and foods; reporting only those with a weighted per cent >5%

^b ^NOS = Not Otherwise Specified

## Discussion

Given the broad scopes of practice of naturopathic medicine in Washington and Connecticut, the similarities in the practitioner characteristics, patient characteristics and practice characteristics in Washington State and Connecticut are remarkable. The finding that naturopathic medicine is primarily a white and female profession is consistent with other recent studies of naturopathic physicians in the United States and Canada [19,20]. Substantial fractions of naturopathic doctors, especially in Washington, are licensed in other CAM and conventional professions. Practitioners spend about 25 hours per week in patient care and see approximately 32 patients/week, which is slightly more than was reported in a recent Canadian study [20]. In comparison, according to the American Medical Association, general practitioners in the United States spend approximately 51.3 hours/week providing direct patient care and see approximately 125 patients in a typical week [16].

Patients who visit naturopathic physicians are primarily female, white, middle aged, non-smokers. In many ways they are demographically similar to the naturopathic physicians themselves. Patients present to naturopathic physicians with a broad range of complaints and are diagnosed by the practitioners with a wide variety of conditions. It should be noted that much of naturopathic practice is focused on chronic problems, particularly those exclusively or primarily presented by women (e.g., fatigue, headache, symptoms associated with menopause, depression). In fact 75% of all visits are made by women. The most common "reasons for visits" identified in this study were very similar to those noted in a Canadian study [20], suggesting that this sample of provider visits may be representative of naturopathic patient visits across North America.

Other than physical examination, blood tests and stool analysis, naturopathic practitioners use few diagnostics tests. However, they commonly recommend commercially packaged natural products such as herbal medicines, vitamins and minerals. Naturopathic practice also involves significant amounts of patient counseling and education. Overall, naturopathic physicians spend more than twice as much time with patients as conventional physicians at each visit (40 minutes vs. 14 minutes) [16], permitting more time to discuss patients' concerns and counseling/education about lifestyle issues such as diet.

Data from this and previous studies [19,20] indicate that patients visit naturopathic physicians for a wide range of conditions and are given a correspondingly wide range of therapies and treatments as part of their naturopathic care. This diversity makes it difficult to describe a "typical" naturopathic medical consultation and to develop standardized research protocols for studies evaluating naturopathic treatments.

Our results also have implications both for naturopathic physicians and the American health care system. They indicate that naturopathic physicians treat a broad range of health problems. The results from our study provide baseline data for tracking changes as the practice of naturopathic medicine evolves over time across the US in response to increasing research into the safety, efficacy and cost-effectiveness of naturopathic treatments, as well as to changes in the social, economic and regulatory environment.

### Strengths and limitations of the study

The major strengths of this study are its large sample size, relatively high participation rates, and the ability to compare naturopathic physicians from two geographically distant states. The main limitation is that despite the similarities of the states studied, it is not known if the results are representative of the other ten states where naturopathic medicine is licensed. These two states reflect typical licensing requirements prevalent throughout the US. Washington State has one of highest numbers of licensed naturopathic physicians in the US, while the number of naturopathic physicians in Connecticut is significantly smaller, and more representative of, most other licensed states.

Another limitation of the survey was its inability to accurately capture the use of office-compounded tinctures and powders. Our results highlight the use of bottled products as a major part of naturopathic practice; however, the practitioners' use of their own combination preparations was difficult to record on the data collection forms. This aspect of practice is likely under-reported in our data.

## Conclusion

Naturopathic physicians and their practices in Washington and Connecticut, two geographically distinct states, are similar. This study provides baseline data for describing and tracking the practice patterns and scope of practice of naturopathic physicians over time and will help inform researchers and policy makers considering the regulation of this profession in other states. It also helps conventional physicians, other health care providers and patients better understand the nature of naturopathic care.

## Competing interests

The authors declare that they have no competing interests.

## Authors' contributions

HB conceptualized and drafted the manuscript. DC conceptualized the overall project, designed and directed collection of the original data and participated in the conceptualization of this manuscript, and analysis of this data. KS participated in the design of the overall project and this manuscript, and participated in the statistical analyses. JE helped to conceptualize the overall project, directed the data collection, quality control, and participated in the analyses for this paper. BM, JB, EC, and DE helped to conceptualize the overall project including the data collection. MS helped to conceptualize this paper. RD helped to conceptualize the overall project, design data collection procedures, and secure funding. All critically edited drafts of this manuscript and approved the final manuscript.

## Pre-publication history

The pre-publication history for this paper can be accessed here:


